# Book Reviews

**Published:** 1906-07

**Authors:** 


### BOOK REVIEWS.

Osborne's Introduction to Materia Medica and Pharmacology. In introduction to the study of Materia Medica and Pharmacology, including the Elements of Medical Pharmacy, Prescription Writing, Medical Latin, Toxicology and Methods of Local Treatment. For the use of students of Medicine and Pharmacy. By Oliver T. Osborne, A.M., M.D., Professor of Materia Medica, Therapeutics and Clinical Medicine in Yale University, ex-President of the American Therapeutic Association, etc. In one 12mo volume of 167 pages. Cloth, $1.00, net. Lea Brothers Co., Publishers, Philadelphia and New York, 1906.

Dr. Osborne is so well known in the field of practical and scientific therapeutics that the appearance of his name on the title page of any book will insure its careful reading. The object of this little book is to introduce the student to the study of Materia Medica and Therapeutics from the most practical standpoint. Every student of medicine, and pharmacy, as well, will find his studies will be made simpler and easier by the preparatory reading of this little volume.

---

The Surgical Pathology and Treatment of Diseases of the Ear. By Clarence John Blake, M.D., Professor of Otology, Medical Department of Harvard University and Henry O. Reik, M.D., Associate in Ophthalmology and Otology, Medical Department Johns Hopkins University. Cloth, $3.50, net. D. Appleton & Co., Publishers, New York.

This work was designed as a guide for the general practitioner and a text-book for the students. "Two efforts have been maintained throughout the book: One to present the subject as simply as possible, the other to eliminate, as far as possible, that obstructive factor in scientific study—personal equation."

The titles of the different chapters give a good idea of the scope of the work.

It begins with a chapter on Surgical Anatomy of the Temporal Bone and Adnexia, following which is a chapter on Aseptic Technique; Chapter III, Diseases of the Auricle and External Auditory Canal; Chapter IV, Diseases of the Tympanic Membrane and Tympanum; Chapter V, The Possible Complications and Consequences of Suppurative Optitis Media; Chapter VI, Middle-Ear Operations; Chapter VII, Mastoid Operations; Chapter VIII, Adventitious Aural Surgery. This chapter treats of a considerable number of operations, which, though not strictly a part of otology, are, however, necessarily connected with the work: Adenoids—their effect upon the organ of hearing. Adenoidectomy—subcutaneous and intravenous infusions—Lumar puncture.

Although the illustrations are not numerous, they are well executed and mostly original. The book is well bound, of convenient size and to be commended for its general excellence.

---

BOVÉE'S GYNECOLOGY. The Practice of Gynecology by Eminent Authorities, edited by J. Wesley Bovée, M.D., Professor of Gynecology in George Washington University, Washington, D.C. In one very handsome octavo volume, containing 838 pages, with 382 engravings and 60 full page plates in colors and monochrome. Cloth, $6.00, net; leather, $7.00, net; half morocco, $8.00 net. Lea Brothers & Co., Publishers, Philadelphia and New York, 1906.

This is the first of a series of three companion volumes dealing, respectively, with Gynecology, Obstetrics and Pediatrics. It is a practical treatise on the diseases of generative organs of women, and on those of the neighboring organs, the urinary system and rectum. The scope has been intentionally made broader than the technical definition of gynecology, with obvious advantages. The work is so arranged that it will be of interest, not only to the gynecologist and surgeon, but also to the large body of practitioners. The illustrations are numerous and excellent, each one clearly proclaiming its message. To secure the advantage of a broad view of the subject, the following members of the medical profession have contributed to the above volume, under the editorship of Dr. Bovée: Dr. J. Riddle Goffe, Dr. G. Brown Miller, Dr. George H. Noble, Dr. Benjamin R. Schenck, Dr. Thomas J. Watkins and Dr. X. O. Werder.

---

DISEASES OF THE NERVOUS SYSTEM RESULTING FROM ACCIDENT AND INJURY. By Pearce Bailey, A. M., M.D., Clinical Lecturer in Neurology, Columbia University, New York City; Consulting Neurologist to the Roosevelt, St. Luke's and Manhattan State Hospitals, etc. D. Appleton & Co., New York and London.

Dr. Bailey is known to the medical world through his former treatise "Accident and Injury in their Relations to the Nervous System," and other contributions to neurology. The present volume begins with a description of the methods of eliciting the essential features regarding the history of the patient, the accident and physical evidence of predisposition to nervous diseases, as well as the examination for the actual injury.

Part I takes up the organic effect of injury to the nervous system. Part II, the functional effect of the injury, and Part III, the medico-legal considerations. All of the subjects are ably dealt with, and the book will undoubtedly win a place for itself as a treatise on all traumatic affections of the nervous system.

The twelve pages of "bibliography" at the end show that the author has made himself thoroughly conversant with the recent literature upon these subjects.

---

We have just received a little booklet on Vibratory treatments, and do not believe we have ever read a more succinct or logical explanation of the value of vibration, and are taking the liberty of quoting from it.

Pathological action is essentially either interrupted or exaggerated physiologic action. All vital processes are now known to be actuated by vibratory motion of one or another velocity.

In a state of health vital vibrations are rhythmic and therefore harmonious. In disease these vibrations are thrown into discord.

Mechanical Vibratory impulses may be made to accelerate vibrations that have been jarred out of rhythm.

What could be more natural than to invoke artificial or mechanical impulses to aid or correct lagging or lacking vital impulses. This indicates the logical and physological basis of mechanical vibration.

It relieves congestion; it equalizes the circulation; it discusses and disperses exudates, tumors, and morbid growths. It soothes perturbed nerves; it allays pain; it rouses dormant nerve centers; it retards and regulates overactive organs or functions; it relaxes contracted and restores atrophied muscles; it is a sovereign and physiologically legitimate remedy in myalgia, lumbago and the various forms of neuralgia.

It is the best known stimulant of absorption and assimilation. It promptly rouses sluggish lymphatics and keeps them in vigorous activity.

In a word, it stimulates in a legitimate and healthful manner the general metabolism of the body; diffusing a glow over the entire system, and imparting a sense of comfort and well being to which the patient has long been a stranger.

Specifically it is applicable to scores of conditions which the foregoing principles will suggest to every thinking physician, and which therefore need not be mentioned.

The publishers are the Sam J. Gorman Co., 153-159 South Jefferson Street, Chicago, and copies will be sent free on request to them.

---

### Early Forthcoming Books.

The following books, by well-known authors, are to be published at an early date by the *Cleveland Press,* Chicago:

*The Organization, Construction and Management of Hospitals,* with numerous plans and details. By Albert J. Ochsner, B. S., F. R. M. S., M.D., and Meyer J. Sturm, B.S., architect, Chicago. The present time may be appropriately termed the *Hospital Age,* and this book is very opportune, as even the smaller cities and towns are now being supplied with sanatoriums, hospitals, etc. In the construction of these there has been a long-felt need for a book by an experienced and representative man as Dr. Ochsner, and from the prospectus before us we have every reason to believe his book will meet with the hearty approval of the profession.

*Practical Dermatology. A Condensed Manual of Diseases of the Skin, Designed for the Use of Students and Practitioners of Medicine.* By Bernard Wolff, M.D., Atlanta, Ga.

Dr. Wolff is favorably known to the medical profession through his association with the Atlanta College of Physicians and Surgeons as Clinical Professor of Diseases of the Skin, and by his able editorship of the ATLANTA JOURNAL-RECORD OF MEDICINE. We look forward with much pleasure to the publication of this manual, as Dr. Wolff has a very terse and attractive style of writing, and has had a large practical experience in his chosen specialty.

*The Technique of Modern Operations for Hernia Operations.* By Alexander Hugh Ferguson, M.B., M.D., Professor of Clinical Surgery, Medical Department of the University of Illinois, etc., and

*A Practical Guidebook on Every-Day Surgery and Surgical Handicraft.* By A. Hamilton Levings, M.D., Professor of the Principles and Practices of Surgery in the Wisconsin College of Physicians and Surgeons, etc.

---

THE design for the memorial to Rudolf Virchow at Berlin which won the first prize represents a titan struggling with a sphinx. This portrays the struggle between Virchow, the giant of science, and the riddle of the origin of disease. The bronze group stands on a marble pedestal which bears Virchow's portrait in a bronze relief.

---

Do not be too hasty in making a diagnosis of intercostal neuralgia. With the exception of pulmonary and pleural conditions, ulcer of the stomach simulates intercostal neuralgia more frequently than any other lesion.—*American Journal of Surgery.*

